# Cytotoxicity of natural and synthetic cannabinoids and their synergistic antiproliferative effects with cisplatin in human ovarian cancer cells

**DOI:** 10.3389/fphar.2024.1496131

**Published:** 2024-11-26

**Authors:** Ying Chen, Huifang Li, Jia Liu, Jie Ni, Qicheng Deng, Haotian He, Panpan Wu, Yinsheng Wan, Navindra P. Seeram, Chang Liu, Hang Ma, Weipei Zhu

**Affiliations:** ^1^ Department of Obstetrics and Gynecology, The Second Affiliated Hospital of Soochow University, Suzhou, China; ^2^ Bioactive Botanical Research Laboratory, Department of Biomedical and Pharmaceutical Sciences, College of Pharmacy, University of Rhode Island, Kingston, RI, United States; ^3^ Department of Operation Room, The Second Affiliated Hospital of Soochow University, Suzhou, China; ^4^ Guangdong Provincial Key Laboratory of Large Animal Models for Biomedicine, School of Pharmacy and Food Engineering, Wuyi University, Jiangmen, China; ^5^ Department of Biology, Providence College, Providence, RI, United States

**Keywords:** cannabinoids, cannabidiol, ovarian cancer, synergistic effect, cisplatin

## Abstract

**Introduction:**

Cannabinoids are reported to suppress the growth of ovarian cancer cells, but it is unclear whether structural modifications can improve their cytotoxic effects.

**Methods:**

Herein, an investigation into the antiproliferative effects of natural cannabinoids on human ovarian cancer Caov-3 cells identified cannabidiol (CBD) as the most promising cannabinoid. Furthermore, chemical modifications of CBD yielded a group of derivatives with enhanced cytotoxicity in Caov-3 cells.

**Results:**

Two CBD piperazinyl derivatives (**19** and **21**) showed augmented antiproliferative effects with an IC_50_ of 5.5 and 4.1 µM, respectively, compared to CBD’s IC_50_ of 22.9 µM. Further studies suggest that modulation of apoptosis and ferroptosis may contribute to the cytotoxic effects of CBD and its derivatives. In addition, CBD and its derivatives (**19** and **21**) were explored for their potential synergistic antiproliferative effects in combination with chemotherapeutic agent cisplatin. Compounds **19** or **21** (5 µM) combined with cisplatin (1 µM) showed a synergistic effect with a combination index of 0.23 and 0.72, respectively. This effect was supported by elevated levels of reactive oxygen species in Caov-3 cells treated with cisplatin combined with **19** or **21**.

**Discussion:**

Findings from this study suggest that CBD derivatives with enhanced antiproliferative effects may exert synergistic effects with chemotherapeutic drugs, providing insight into the development of cannabinoid-based adjuvant agents for the management of ovarian cancer.

## Graphical Abstract

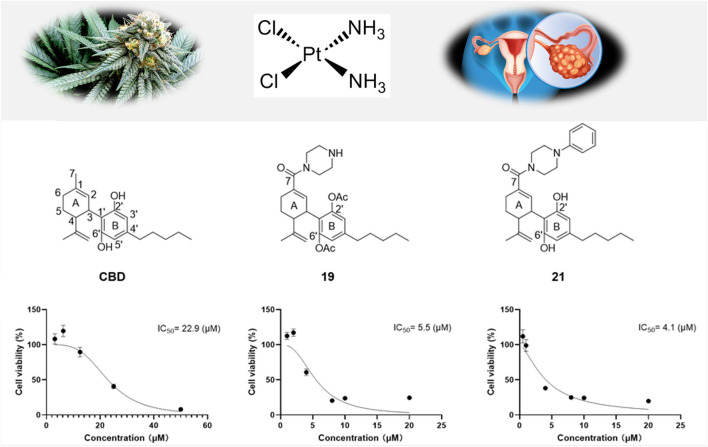

## 1 Introduction

Ovarian cancer is a malignancy originating in the ovarian epithelium. The etiology of ovarian cancer involves complex genetic, environmental, and hormonal factors, making it a multifaceted disease (Jayson et al., 2014). Ovarian cancer is the fifth leading cause of cancer-related deaths among women, highlighting the unmet medical need for effective therapeutic strategies. Current treatment typically involves a combination of surgical intervention and chemotherapy (Cortez et al., 2018). The standard chemotherapy approach heavily relies on platinum-based compounds (such as cisplatin and carboplatin), which exert anti-cancer effects by inducing DNA cross-linking and subsequent apoptosis in cancer cells. However, the efficacy of these treatments is often hampered by the high incidence of chemoresistance and significant adverse effects (Zoń and Bednarek, 2023). Thus, exploring alternative or adjunctive therapeutic options is critical to improving chemotherapeutic outcomes. Natural products have been reported for their antiproliferative effects against ovarian cancer cells through various mechanisms including apoptosis induction, cell cycle arrest, and inhibition of angiogenesis (Pistollato et al., 2017). Additionally, natural products studied in combination with conventional chemotherapeutic agents (i.e., cisplatin) show promise in enhancing anti-cancer effects and overcoming drug resistance. Synergistic interactions between natural compounds and cisplatin can potentiate the efficacy of treatment by targeting multiple cellular pathways simultaneously (Dasari et al., 2022). For instance, curcumin, a polyphenol from turmeric, has been shown to sensitize ovarian cancer cells to cisplatin, enhancing its cytotoxic effects (Montopoli et al., 2009). Such combinatorial approaches aim not only to reduce the dosage and side effects of chemotherapeutic drugs but also to achieve more comprehensive and sustained anti-cancer responses. This integrative strategy supports the potential use of natural products in conjunction with conventional therapies to develop more effective treatments for ovarian cancer.

Cannabinoids are a group of structurally diverse bioactive phytochemicals from Cannabis. These natural products can exert biological effects through the endocannabinoid system (ECS), which regulates cell signaling pathways that modulate cell survival, migration, and invasion (I. Khan et al., 2016; Heider et al., 2022) Notably, components of the ECS, such as cannabinoid receptor 1 (CB1), CB2, and fatty acid amide hydrolase, are expressed in normal human ovaries (El-Talatini et al., 2009). Moreover, the levels of ECS ligands, including anandamide, N-oleoylethanolamine, and N-palmitoylethanolamine, are exacerbated in the follicular fluids of ovarian cancer patients (Schuel et al., 2002). These studies suggest a possible involvement of ECS in the development of ovarian cancer. As exogenous ligands of the ECS, natural cannabinoids such as cannabidiol (CBD; a major non-psychedelic phytocannabinoid in Cannabis) and cannabigerol (CBG; a precursor of CBD) are reported to induce selective cytotoxicity in both cisplatin-sensitive and cisplatin-resistant ovarian cancer cells (Sooda et al., 2023). Natural cannabinoids have different chemotypes, such as CBD, CBG, cannabinol (CBN), and Δ8-tetrahydrocannabinol (Δ8-THC). These cannabinoids have distinct structural characteristics, including differences in alkyl side chains, aromatic ring structures, and hydroxyl group substitutes, which may impose varying affinities for ECS and other pharmacological profiles. In addition, the antiproliferative effects of CBD and CBG are associated with the modulation of apoptosis and elevated reactive oxygen species (ROS) in mitochondria. Moreover, these cannabinoids have been studied for potential anti-cancer properties (Jastrząb et al., 2022; Ledvina et al., 2023; ALSalamat et al., 2024). However, it remains unclear whether other natural cannabinoids can exert antiproliferative effects against ovarian cancer cells, or whether chemical modifications of natural cannabinoids can result in augmented anti-cancer activity.

Thus, our group has initiated a research program to evaluate the biological activities of natural cannabinoids and their synthetic analogs (Liu et al., 2020; Liu et al., 2021; Ma et al., 2021; Puopolo et al., 2023; Zhang et al., 2023; Li et al., 2024; Lyu et al., 2024). During this investigation, CBD was identified as a lead compound with a moderate antiproliferative effect on melanoma cells (Lyu et al., 2024). Furthermore, a library of CBD derivatives was synthesized and assayed to study the structure-activity relationships, revealing that CBD analogs with a bipiperidinyl group significantly enhanced the antiproliferative effects (Lyu et al., 2024). However, it remains unknown whether the modification of cannabinoids can improve their inhibitory effects on the growth of ovarian cancer cells. Herein, we sought to evaluate the antiproliferative effects of a panel of natural and synthetic cannabinoids and explore the mechanisms of action. Additionally, combinations of cannabinoids and cisplatin at various concentrations were evaluated for synergistic antiproliferative effects, which is critical for the potential development of cannabinoid-based adjuvant agents.

## 2 Experiment design

### 2.1 Cell line

The human ovarian cancer cell line (Caov-3 cells) was purchased from the American Type Culture Collection (ATCC; Rockville, MD, United States) and cultured with Dulbecco’s Modified Eagle’s Medium (DMEM; Thermo Fisher Scientific, Waltham, MA, United States) with 10% fetal bovine serum (FBS; Thermo Fisher Scientific) and 1% penicillin/streptomycin (Sigma-Aldrich Co., St. Louis, MO, United States). The cells were *mycoplasma* negative and incubated at 37°C with 5% CO_2_.

### 2.2 Chemicals and reagents

Natural phytocannabinoids, including cannabichromene (CBC), cannabidiol (CBD), cannabigerol (CBG), cannabinol (CBN), cannabicitran (CBT), cannabidiolic acid (CBDA), cannabidivarin (CBDV), cannabigerolic acid (CBGA), and delta-8-tetrahydrocannabinol (Δ8-THC), were purchased from Cayman Chemical (Ann Arbor, MI, United States). A collection of CBD derivatives was chemically synthesized by our laboratory following a previously reported protocol (Zhang et al., 2023) Cannabinoids were dissolved in dimethyl sulfoxide (DMSO; Thermo Fisher Scientific) at 100 mM and stored in aliquots at −20°C. Cisplatin was purchased from Sigma-Aldrich (St. Louis, MO, United States) and a FerroOrange (#F374) assay kit was purchased from Dojindo Lab (Kumamoto, Japan). Paraformaldehyde, phosphate-buffered saline (PBS; pH = 7.4), [4,5-Dimethyl-2-thiazolyl]-2,5-diphenyltetrazolium bromide (MTT), and the Annexin-V (FITC) apoptosis detection kit were purchased from Thermo Fisher Scientific (Waltham, MA, United States).

### 2.3 Cell viability assay

The MTT assay was performed to evaluate the viability of Caov-3 cells. The cells were seeded in 96-well plates at a density of 5,000 cells per well and incubated for 24 h at 37°C and 5% CO_2_. After treatment of various cannabinoids or combinations of cannabinoid and cisplatin (added simultaneously in the synergistic assays) for 24 or 48 h, cell culture medium was replaced with 100 μL of fresh medium containing MTT reagent (10 μL) and incubated for 4 h. Then culture medium was discarded and replaced with 100 μL of DMSO to solubilize formazan crystals and the absorbance of each well was recorded by a plate reader (SpectraMax M2; Molecular Devices, Sunnyvale, CA, United States) at 570 nm. The synergistic effects of combination treatments were assessed by the combination index (CI) values obtained from the Compusyn Software (www.combosyn.com) (Li et al., 2022).

### 2.4 Colony formation assay

The cells were seeded in 6-well plates (2 mL/well) at a density of 1,000 cells per well and incubated overnight. After the treatment with test samples for 48 h, the medium was replaced with fresh medium every three days and cultured for 10–14 days. The cells were washed twice with PBS and then fixed with 4% paraformaldehyde for 20 min. The plates were stained with 0.1% crystal violet for 20 min at room temperature. Then, the cells were washed with PBS three times, dried upside down, and the colonies’ images were finally recorded and analyzed by ImageJ (http://rsb.info.nih.gov/ij/).

### 2.5 Cellular iron assay

The FerroOrange staining assay was used to detect the content of intracellular ions (Fe^2+^) using a reported method (Li et al., 2022). Caov-3 cells were seeded in 6-well plates (2 mL/well) at a density of 400,000 cells per well and incubated overnight. Then, the cells were treated with different concentrations of test compounds with or without cisplatin (1 μM) for 48 h. Cells were then collected and incubated with FerroOrange agent in the dark at 37°C for 30 min. The cellular fluorescence was analyzed by flow cytometry (BD FACSVerse; San Jose, CA, United States). A total of 10,000 events were acquired for each sample from three independent experiments. Fluorescence was measured by using FlowJo software.

### 2.6 Cell apoptosis measurements

An Annexin V-fluorescein isothiocyanate (FITC)/propidium iodide (PI) apoptosis kit (Thermo Fisher Scientific, Waltham, MA, United States) was used to detect the percentage of cell apoptosis. Cells were incubated in 6-well plates (2 mL/well) with a density of 400,000 per well for 24 h, followed by treatment with compounds with or without cisplatin (1 μM) for 48 h. All cells were then resuspended in pre-chilled 1× binding buffer, stained with FITC and PI for 15 min at room temperature in the dark, and analyzed by a flow cytometer.

### 2.7 Measurement of intracellular reactive oxygen species

A fluorescent probe (2′,7′-dichlorofluorescin diacetate; DCF-DA; Sigma-Aldrich, St. Louis, MO, United States) was used to detect the amounts of intracellular reactive oxygen species (ROS). The cells were incubated in 6-well plates (2 mL/well) at a density of 150,000 per well for 24 h, followed by treatment with test compounds with or without cisplatin (1 μM) for 48 h. Cells were centrifuged and incubated with DCF-DA (20 μM) at 37°C for 30 min in the dark and analyzed by a flow cytometer.

### 2.8 Statistics

The data collected was presented as mean value ±standard deviation (SD) and analyzed by GraphPad Prism (Version 10.0; GraphPad Software, La Jolla, CA, United States). The significance of differences between groups was determined using a one-way analysis of variance (ANOVA). Differences were considered to be statistically significant with *p < 0.05, **p < 0.01 and ***p < 0.001.

## 3 Results and discussion

### 3.1 The cytotoxic effect of natural cannabinoids in ovarian cancer cells

**FIGURE 1:**
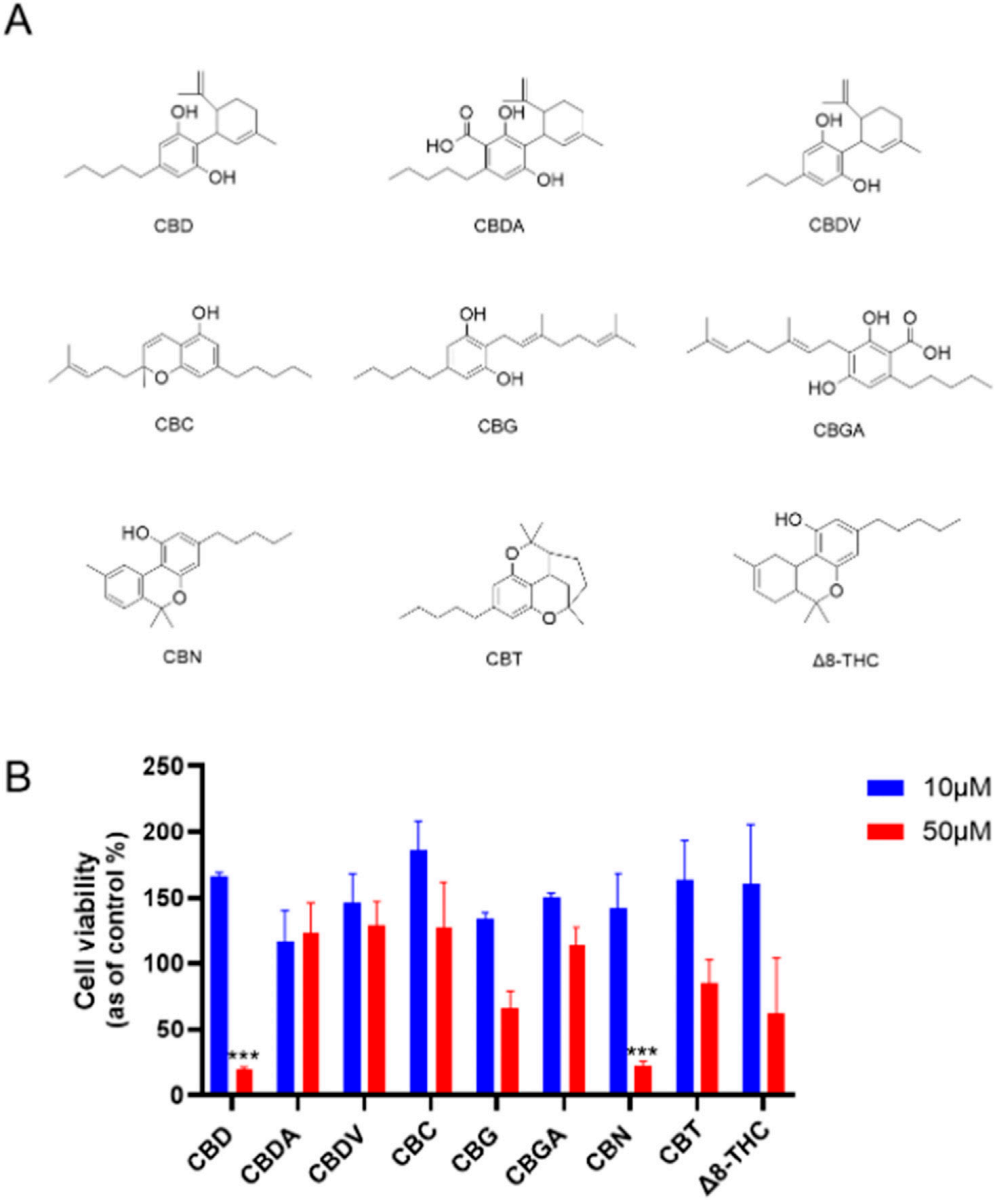
**(A)** Chemical structures of natural cannabinoids. **(B)** Effects of natural cannabinoids on the viability of Caov-3 cells at 10 and 50 µM.

First, the cytotoxicities of natural cannabinoids including CBD, CBDA, CBDV, CBC, CBG, CBGA, CBN, CBT, and Δ8-THC (Chemical structures shown in Figure 1A) were evaluated in a human ovarian cancer cell line (Caov-3 cells). At a lower concentration (10 µM), natural cannabinoids had no inhibition on the growth of Caov-3 cells. Two cannabinoids, namely, CBD and CBN, showed significant cytotoxicity at a higher concentration (50 µM) with a cell viability of 19.9% and 22.7%, respectively, while other natural cannabinoids including CBG, CBT, and Δ8-THC showed weak inhibition (<50%) (Figure 1B).

Our study extends previous research that has explored the anticancer properties of cannabinoids, particularly focusing on CBD, CBG, CBN, and Δ8-THC. Reported studies have shown that these compounds can induce cell death in various cancer types through mechanisms such as apoptosis, autophagy, and oxidative stress induction. For instance, CBD has been well-documented to exert antiproliferative effects by promoting apoptosis and increasing reactive oxygen species (ROS) in cancer cells, including ovarian and breast cancer models (Ma et al., 2023; Sooda et al., 2023). Similarly, CBG and CBN have shown cytotoxic potential, despite with less comprehensive data than CBD, suggesting further mechanistic exploration of these cannabinoids in cancer therapy is needed. Our study extends this body of research by evaluating natural cannabinoids, which identified CBD as a lead compound with higher cytotoxicity in Caov-3 ovarian cancer cells than other natural cannabinoids.

Although cannabis has been empirically used to treat various cancers, including ovarian cancer, clinical trials have not supported its effectiveness in treating ovarian cancer. To date, only a case study has reported that treatment of “CBD oil” decreased the size of the bilateral adnexal masses and mesenteric and pelvic lymphadenopathy in one patient (Barrie et al., 2019). Similarly, preclinical studies on the anti-cancer properties of cannabis or its compounds are limited. Several *in vitro* studies showed that CBD can inhibit the growth of ovarian cancer cells (including A2780, A2780/CP70, SKOV3, and Caov-3 cells) by inducing apoptosis (Ma et al., 2023; Sooda et al., 2023). Additionally, less studied cannabinoids, such as CBG and CBN, have shown cytotoxicity in various ovarian cancer models, suggesting they may also hold potential for further development (Ma et al., 2023). Δ8-Tetrahydrocannabinol (Δ8-THC), which is an isomer of Δ9-THC, has displayed unique effects that may offer therapeutic benefits without the psychoactive properties of Δ9-THC (Mangal et al., 2021). However, the cytotoxicity of other cannabinoids in ovarian cancer cells remains unclear. Consequently, we expanded our biological evaluations from CBD to other natural cannabinoids. Our studies have shown that CBD and CBN have superior antiproliferative effects compared to other cannabinoids, suggesting their potential as parent compounds for the development of synthetic cannabinoids with enhanced anti-cancer activity.

### 3.2 The cytotoxic effect of synthetic cannabinoids in ovarian cancer cells

**FIGURE 2:**
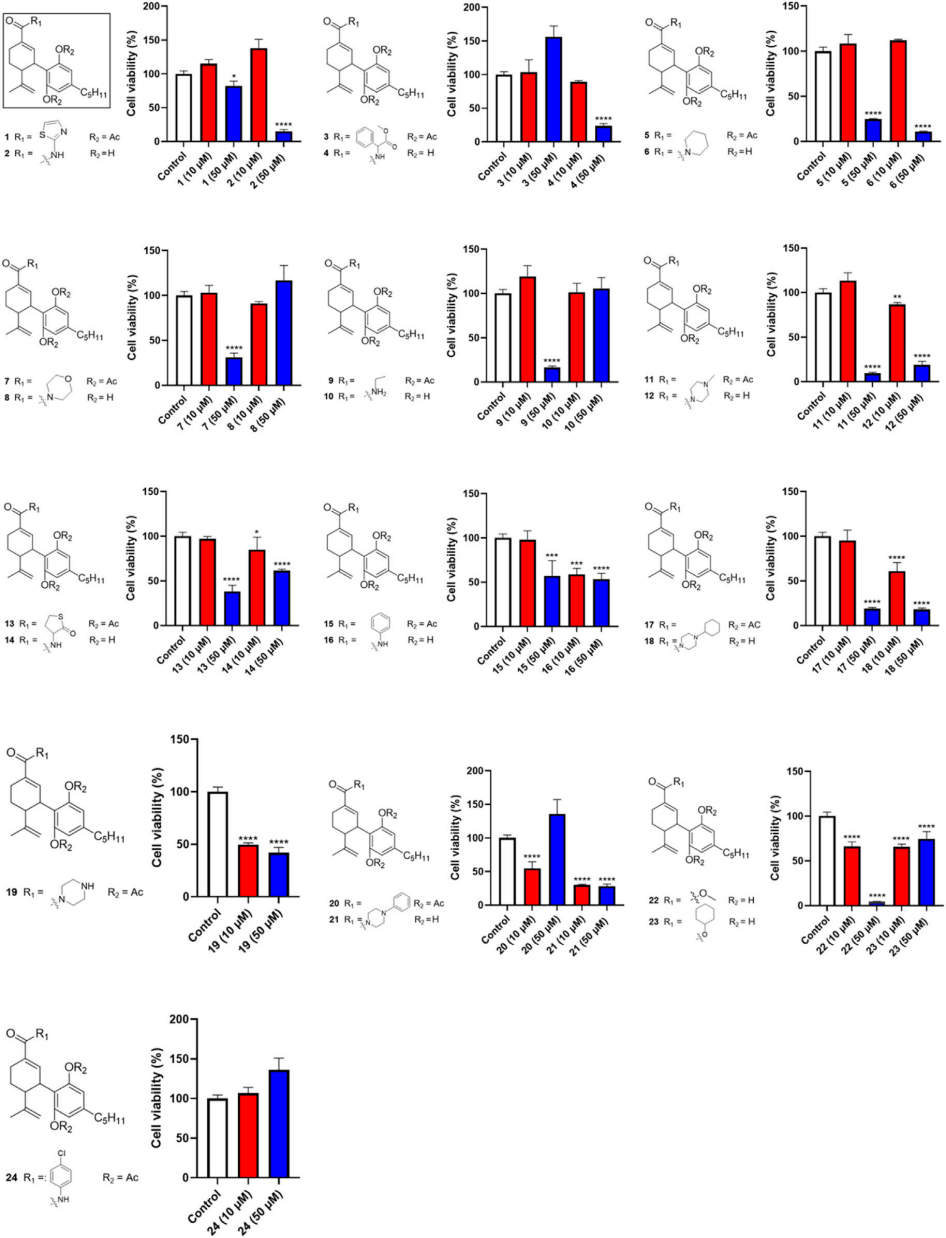
Effects of synthetic cannabinoids **(1–24)** at concentrations of 10 and 50 µM on the viability of Caov-3 cells.

Given that CBD showed the most promising antiproliferative effect in Caov-3 cells, a library of synthetic CBD analogs (**1**–**56**) was further evaluated for their cytotoxicity. The first group of CBD analogs features various substituent moieties at the −7 position of CBD via oxidation and the -2′ or -6′ positions via acetylation. At a higher concentration (50 µM), several analogs showed enhanced antiproliferative effects compared to CBD (Figure 2). Notably, a few compounds remained cytotoxic at the lower concentration (10 µM). For instance, Compound **19**, which has a N-methylpiperazine moiety at the −7 position and two acetate groups at the -2′ and -6′ positions, reduced the viability of Caov-3 cells to 41.9% and 49.6% at 50 and 10 µM respectively. When the N-methylpiperazine group was modified to obtain an analog with a phenylpiperazine group (compound **20**), the antiproliferative effect was comparable (cell viability = 54.6%) at 10 µM. Furthermore, the antiproliferative activity of compound **21** (as the two acetate moieties of compound **20** were replaced by hydrogen) improved to 30.0% at 10 µM.

**FIGURE 3:**
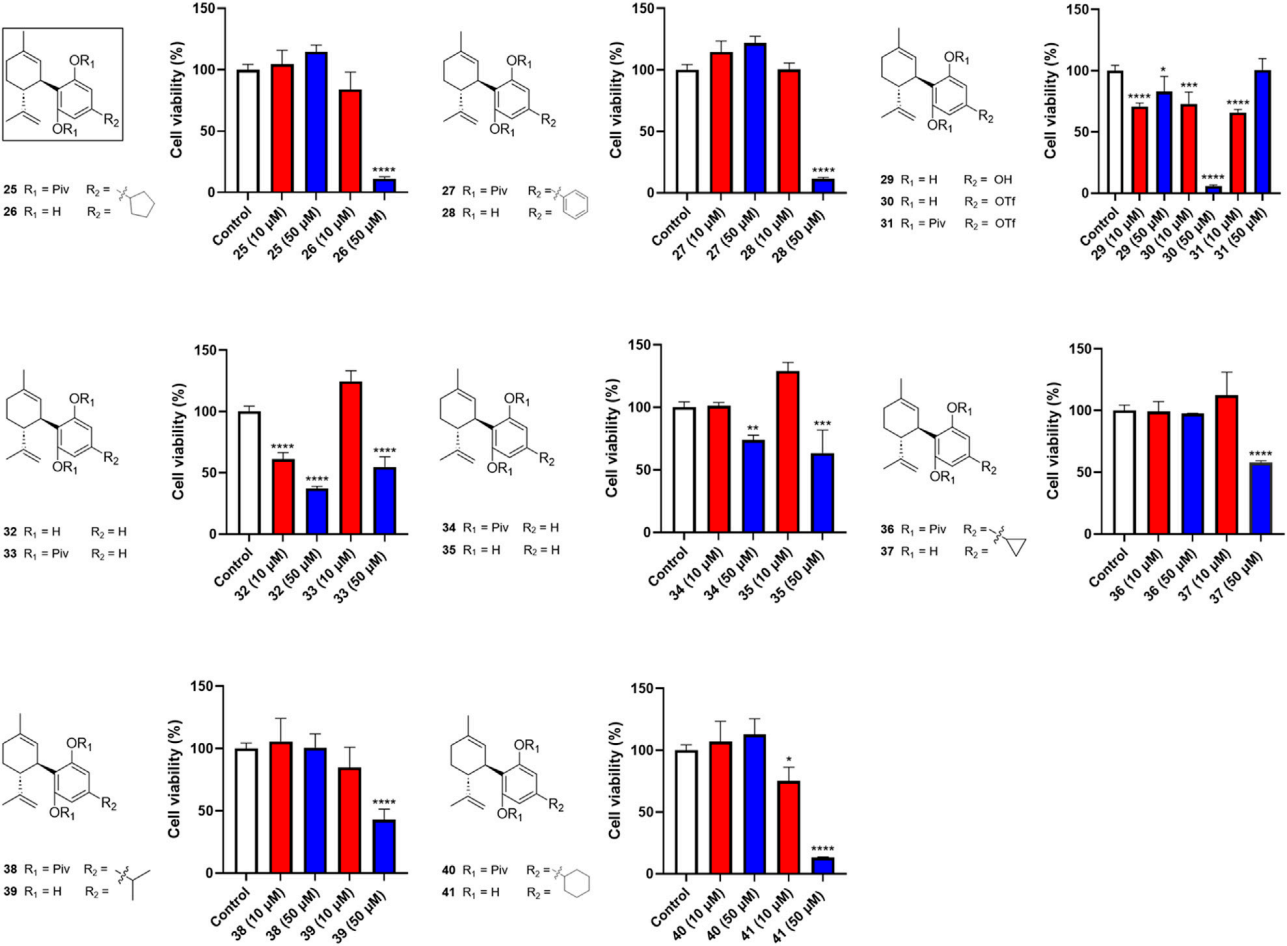
Effects of synthetic cannabinoids **(25–41)** at concentrations of 10 and 50 µM on the viability of Caov-3 cells.

Next, we evaluated the impact of various side chains at the -4′ position of CBD on its antiproliferative effect. As shown in Figure 3, this group of CBD analogs (**25–41**) exhibited moderate cytotoxicities. Compound **26** (with a side chain of a cyclopentyl group), compound **28** (with a side chain of a phenyl group), and compound **41** (with a side chain of a cyclohexyl group) reduced the viability of Caov-3 cells to 11.2%, 11.5%, and 13.2%, respectively, at the concentration of 50 µM. Notably, a reaction intermediate (compound **30**) with a Triflate group (-OTf) had a strong antiproliferative effect on cell viabilities of 5.9% at the higher concentration of 50 µM. However, its cytotoxicity diminished at lower concentrations (10 μM; cell viability = 72.9%).

**FIGURE 4:**
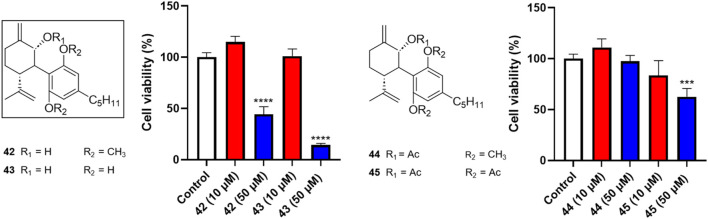
Effects of synthetic cannabinoids **(42**–**45)** at concentrations of 10 and 50 µM on the viability of Caov-3 cells.

Four analogs (**42–45**) with chemical modifications at the oxidized −2 position of CBD were also evaluated for their antiproliferative effects in Caov-3 cells. Only two compounds (**42** and **43**), which had an -OH group at the −2 position and methyl or -H at the -2′ and -6′ positions respectively showed cytotoxicities at the concentration of 50 µM (cell viability = 44.3% and 14.3%, respectively). Other analogs in this group were not active (Figure 4).

**FIGURE 5:**
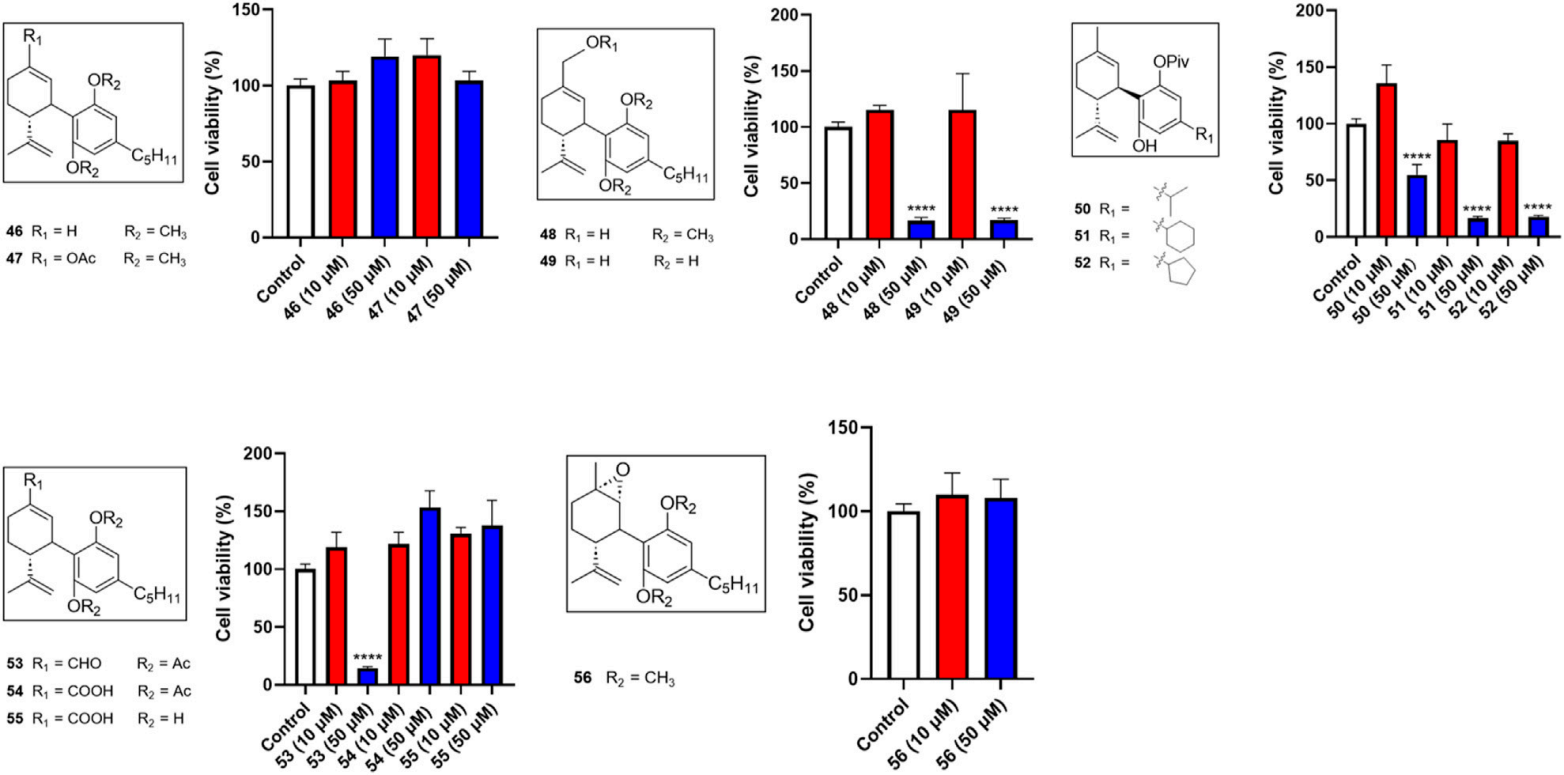
Effects of synthetic cannabinoids **(46**–**56)** at concentrations of 10 and 50 µM on the viability of Caov-3 cells.

Several miscellaneous analogs with various chemical modifications were also evaluated in the MTT assay. Compounds **48** and **49**, analogs with a methanol group at the −7 position of CBD and a -OCH3 or -OH group at the -2′ and -6′ positions respectively, showed cytotoxic effects at 50 µM with cell viabilities of 16.4% and 16.9%, respectively. Compound **53** (with an aldehyde group at the 7-position and diacetate groups at the -2′ and -6′ positions) had a comparable effect with a cell viability of 14.5% at a concentration of 50 µM. Several reaction intermediates, including **51** and **52** (with a pivalic acid group; -OPiv), had similar cytotoxicity at 50 µM with cell viability of 16.4% and 18.0%, respectively (Figure 5).

Our biological evaluations support the notion of enhanced antiproliferative effects by modifications of CBD. This is consistent with our previously reported data showing that introducing a bipiperdinyl moiety to CBD can enhance its antiproliferative effect in melanoma cells (Lyu et al., 2024). In the current study, stronger antiproliferative effects were observed with CBD analogs containing a piperazine moiety, a common functional group used in medicinal chemistry. Numerous molecules with a piperazine group have been extensively investigated for a wide range of therapeutic effects, including anti-cancer activity (Rathi et al., 2016). Several clinically used anti-cancer drugs, such as Imatinib, Everolimus, and Irinotecan, contain piperazine pharmacophores. Notably, it has been reported that incorporation of piperazinyl groups into a natural product, apigenin, greatly improved the antiproliferative effects of its derivatives in human ovarian cancer cells (SK-OV-3) in both *in vitro* and *in vivo* models (Long et al., 2021). This study suggests that the enhanced anti-cancer effects of the piperazinyl derivatives of apigenin are attributed to their chemico-physical properties, such as the improved drug-likeness scores. However, the drug-likeness scores of CBD, **19**, and **21** calculated by a quantitative estimation of drug-likeness (QED) approach were similar, with a drug-likeness value of 0.51, 0.23, and 0.34, respectively. Thus, additional experiments are warranted to elucidate how piperazine moiety increased the antiproliferative activity of CBD. Nevertheless, CBD piperazinyl derivatives showed stronger growth inhibitory effects in Caov-3 cells but further characterizations of their antiproliferative effects are needed.

### 3.3 CBD and its derivatives exert antiproliferative effects in ovarian cancer cells by modulating programmed cell death

**FIGURE 6:**
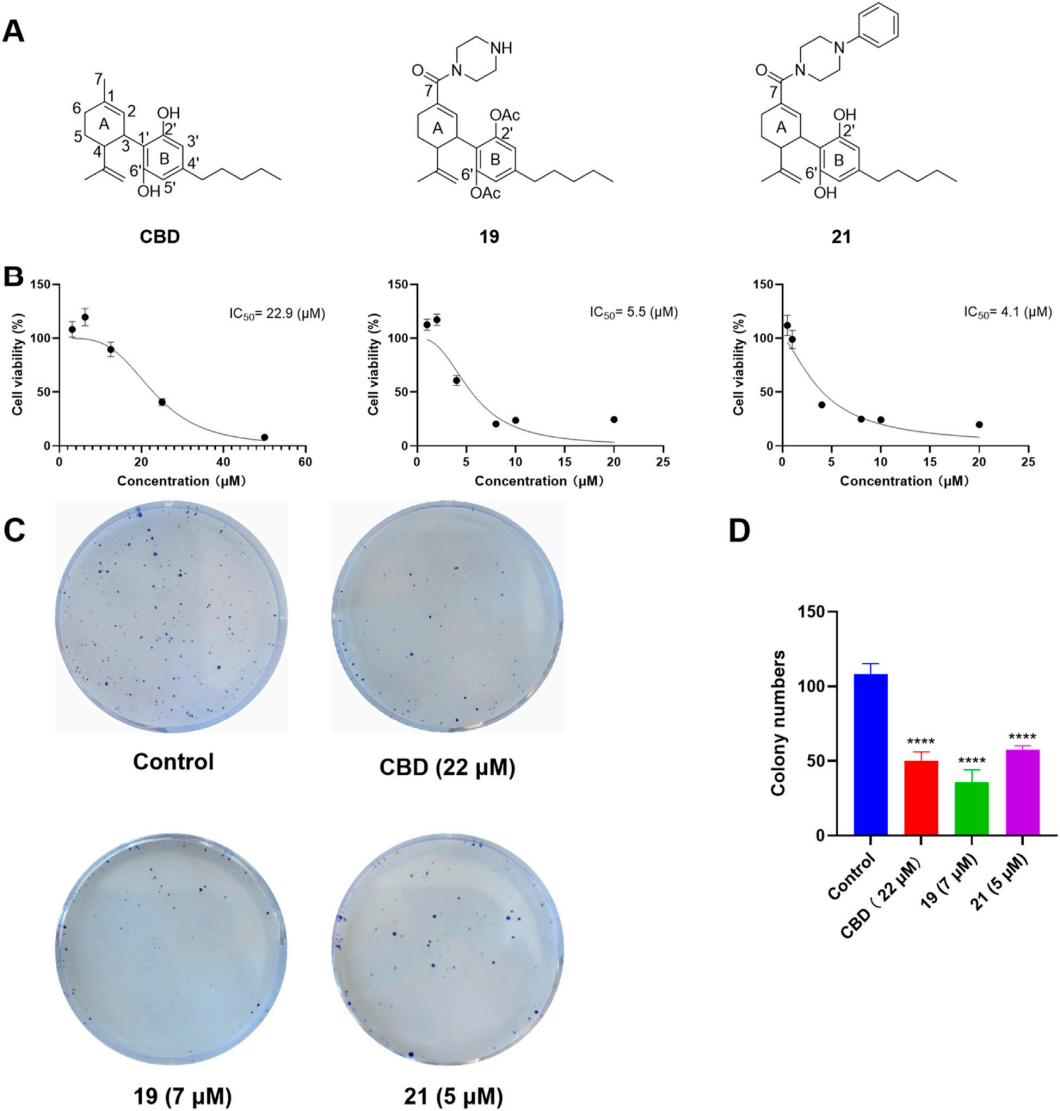
Chemical structure of CBD and its derivatives **19** and **21 (A)**, and their antiproliferative effects against the growth of ovarian cancer Caov-3 cells **(B)**. Clone formation assay in Caov-3 cells treated with CBD, **19** and **21** using crystal violet staining **(C)**. Quantitative analysis of colony numbers treated with CBD, **19**, and **21 (D)**.

Given that compounds **19** and **21** (at 10 and 50 µM) were the most active CBD derivatives in the antiproliferative assay, their inhibitory effects on cell growth were further characterized. The antiproliferative effects of compounds **19** and **21** were enhanced compared to CBD (with an inhibition IC_50_ of 5.5, 4.1, and 22.9 µM respectively; Figure 6B). This effect was supported by a colony formation assay, where treatment with CBD, **19**, and **21** at concentrations near their IC_50_ (i.e., 22, 7, and 5 μM; for 24 h) suppressed the formation of cell colonies by 53.4%, 67.0%, 46.6% respectively (Figures 6C, D).

**FIGURE 7:**
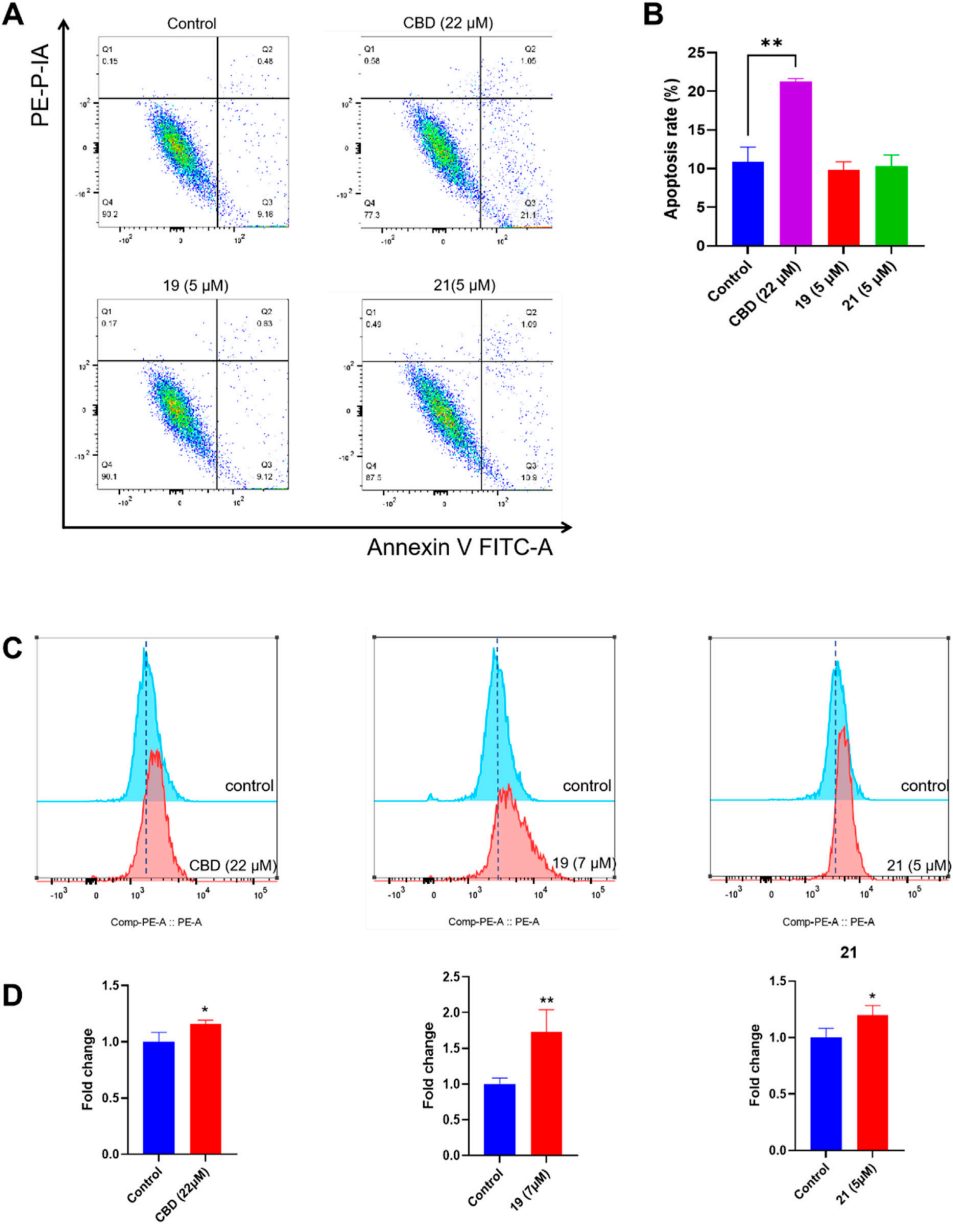
Biological evaluations of apoptosis and ferroptosis in Caov-3 cells exposed to CBD, **19**, and **21**. Flow cytometry dot plots of CBD (22 μM), **19** (5 μM), and **21** (5 μM) -induced apoptosis in Caov-3 cells **(A)**, and the changes in the percentage of apoptosis rate **(B)**. Flow cytometry analysis of intracellular iron by using FerroOrange staining reagent, cells were treated by CBD (22 μM), **19** (7 μM), and **21** (5 μM) **(C)**, and their fold changes in cellular iron level by quantifying the fluorescence signal **(D)**.

The cytotoxicity of CBD and compounds **19** and **21** was further explored by assessing their effects on programmed cell death in Caov-3 cells. We first evaluated whether CBD and its derivatives can promote apoptosis using a flow cytometry assay. Treatment with CBD (22 µM) increased the population of apoptotic cells, whereas **19** (5 µM) and **21** (5 µM) did not show significant effects (Figures 7A, B). Furthermore, these compounds were assayed for biomarkers related to ferroptosis, an iron-dependent form of programmed cell death. Flow cytometry assays can effectively detect ferroptosis by measuring specific cellular markers associated with this iron-dependent cell death pathway. In the current study, we used the FerroOrange assay, which selectively measures ferrous ions (Fe^2^⁺) using flow cytometry, to assess intracellular iron levels. In a typical assay, increased iron levels, indicated by elevated fluorescent signal in the flow cytometric assay, suggest enhanced iron accumulation in cells undergoing ferroptosis. Treatment with CBD (22 µM) and **21** (5 µM) increased the level of cellular iron in Caov-3 cells by 15.7% and 19.9% respectively. The most significant increase in cellular iron level was observed with compound **19**, which elevated the iron level by 73.0% (Figures 7C, D).

Our study provided insights into how CBD and its synthetic derivatives exert cytotoxic effects on ovarian cancer cells, with a focus on apoptosis and ferroptosis as key programmed cell death pathways. Apoptosis, a well-known mechanism of cannabinoid action, is characterized by cellular events such as DNA fragmentation, caspase activation, and cell membrane changes that lead to controlled cell death. Data from these bioassays suggest that the antiproliferative effects of CBD are associated with the induction of apoptosis in Caov-3 cells, which is consistent with previously reported studies showing that CBD promotes apoptosis in cancer cells (Ma et al., 2023; Sooda et al., 2023). However, Compounds **19** and **21** did not increase the population of apoptotic cells, suggesting that these analogs and CBD inhibit cell growth via different mechanisms. Besides apoptosis, other types of programmed cell death may also contribute to the antiproliferative effects of the piperazinyl derivatives of CBD. Compounds **19** and **21** increased the cellular iron level in Caov-3 cells, suggesting that ferroptosis may be a possible mechanism for these CBD derivatives. Interestingly, it has been reported that the antiproliferative effect of erastin, a known ferroptosis inducer developed from cancer research, was improved by the induction of a piperazine moiety in ovarian cancer OVCAR8 cells (Frye et al., 2023). This improvement was attributed to the piperazine moiety enhancing the water solubility and metabolic stability of erastin (Wang et al., 2022). Additional measurements of ferroptosis-related biomarkers, such as SLC7A11 (cystine/glutamate transporter), cellular antioxidant GSH (glutathione), and lipid peroxidation, are needed to confirm whether Compounds **19** and **21** specifically induced ferroptosis in Caov-3 cells. Nevertheless, the distinctive patterns of CBD and its piperazinyl derivatives in inducing programmed cell death provide a rationale for combining cannabinoids with chemotherapeutic agents to exert synergistic effects.

### 3.4 CBD and its derivatives exert synergistic antiproliferative effect with cisplatin

**FIGURE 8:**
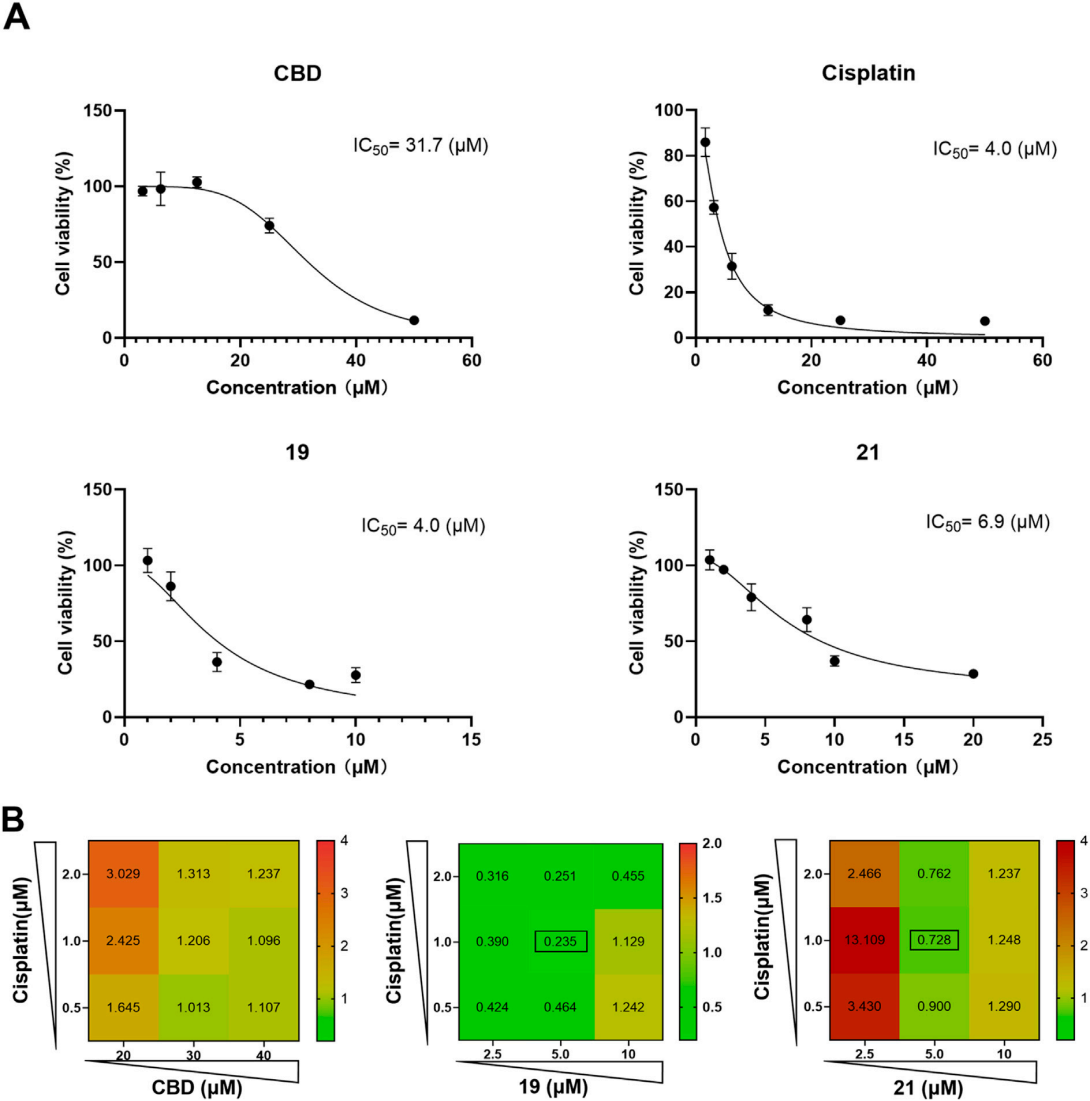
The antiproliferative effects of cisplatin, CBD, **19** and **21** against the growth of ovarian cancer Caov-3 cells and their synergistic effects. The antiproliferative IC_50_ of cisplatin, CBD, and compounds **19** and **21** for a treatment period of 48 h **(A)**. The combination index of CBD, **19**, **21** with cisplatin in various concentrations **(B)**.

Although chemical modifications improved the cytotoxicity of CBD in Caov-3 cells, these cannabinoids may not stand alone as anti-cancer drugs (Huang et al., 2021). Interestingly, CBD has been reported to exert synergistic effects together with cancer chemotherapeutics, such as cisplatin, to suppress the growth of various cancer cells including melanoma, non-small cell lung, bladder, and head and neck squamous carcinoma cells (Go et al., 2020; Misri et al., 2022; Whynot et al., 2023). Therefore, we further evaluated whether CBD derivatives **19** and **21** can enhance the antiproliferative effect of cisplatin in Caov-3 cells. First, the antiproliferative IC_50_ of cisplatin, CBD, and compounds **19** and **21** for a treatment period of 48 h was assessed. Cisplatin, **19**, and **21** had a similar cytotoxic effect with an IC_50_ of 4, 4, and 6.9 µM, respectively, whereas CBD showed a weaker effect (IC_50_ = 31.7 µM; Figure 8A). Next, the synergistic effects of CBD, **19**, and **21** in combination with cisplatin at various concentrations were evaluated by determining their combination index (CI). When the CI is less than 1, it suggests that the combinations exert synergistic effects, whereas a CI greater than 1 indicates antagonistic effects. CBD did not enhance the cytotoxicity of cisplatin as its CIs were greater than 1 (Figure 8B). Compound **19** (at 2.5, 5, and 10 µM) in combination with cisplatin (0.5, 1, and 2 µM) showed promising synergistic effects with CIs less than 1. The most synergistic effect was observed in the combination of 1 µM of cisplatin and 5 µM of **19** (CI = 0.235). Compound **21** showed synergistic effects at 2.5, 5, and 10 µM with cisplatin (CIs in a range from 0.7 to 0.9).

**FIGURE 9:**
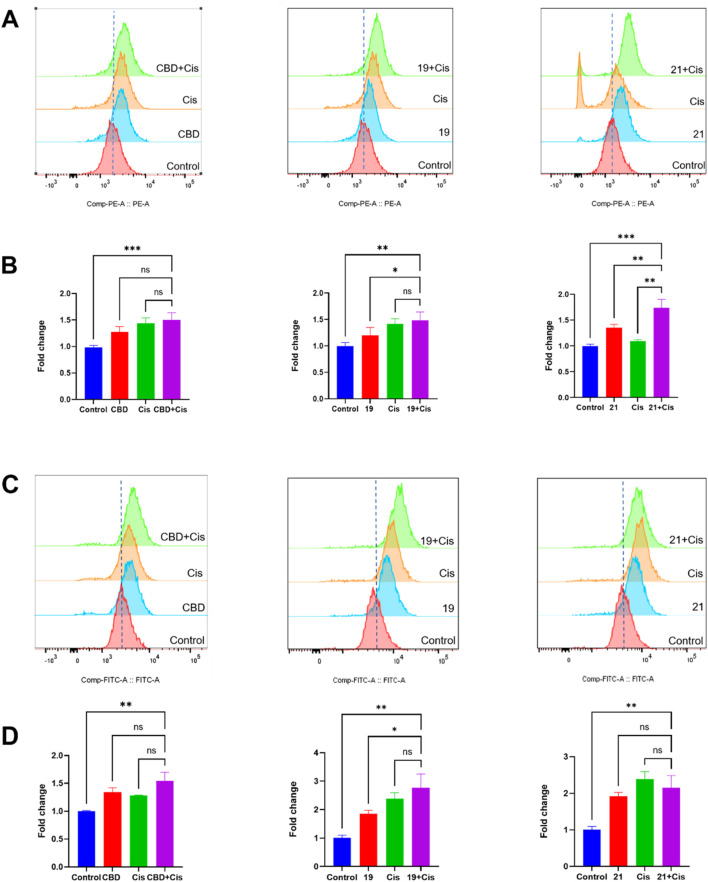
The synergistic effects of cellular iron and ROS levels in Caov-3 cells exposed to CBD, **19**, **21** combined with cisplatin for a treatment period of 48 h. Flow cytometry analysis of intracellular iron in Cao3 cells, cells were treated by CBD (15 μM),**19** (5 μM) with cisplatin (1 μM), **21** (5 μM) with cisplatin (2.5 μM) using the FerroOrange staining reagent **(A)**, and quantitative analysis of iron level detected by fluorescence signals **(B)**. Flow cytometry analysis of ROS by using DC-FDA reagent, cells were treated by CBD (22 μM), **19** (5 μM), **21** (5 μM) with cisplatin (1 μM) **(C)**, and the fold changes quantified by fluorescence signal **(D)**.

We further explored biomarkers (cellular iron and ROS levels) that may contribute to the observed synergistic effects. In the FerroOrange assay, compounds **19** or **21** combined with cisplatin showed elevated iron levels in Cavo-3 cells by 49.2% and 74.1% respectively (Figures 9A, B). Although the combinations increased the ROS level compared to cells in the control group, only those treated with the combination of **19** and cisplatin showed a significant increase of ROS by 48.9% when compared to treatment with compound **19** alone (Figures 9C, D).

Several studies have shown that CBD and cisplatin can confer synergistic effects in cancer cells. For instance, co-treatment with CBD and cisplatin promoted cell death in head and neck squamous cell carcinoma (HNSCC) in cellular assays and a xenograft animal model (Go et al., 2020). Moreover, CBD and carboplatin (a less toxic analog of cisplatin) exerted a synergistic effect that selectively inhibited the growth of ovarian cancer A2780 cells (Sooda et al., 2023). However, CBD and cisplatin (at the tested concentration range and timepoint) had no synergistic effect against the growth of Caov-3 cells, suggesting that further optimization of the dosage is warranted to pursue their synergistic effects. The combinations of compound **19** and cisplatin showed strong synergistic effects with CIs in a range of 0.1–0.3 (Bijnsdorp et al., 2011). The mechanism underlying this synergy may involve the enhanced generation of ROS, mitochondrial dysfunction, and iron dysregulation, as observed in our study. The combination treatment also amplified oxidative stress and cellular iron levels, which are key factors in programmed cell death pathways like apoptosis and ferroptosis. However, treatment of Compound **19** and cisplatin did not significantly increase the iron and ROS levels in Caov-3 cells. Notably, an opposite effect was observed where Compound **21** and cisplatin effectively increased cellular iron but showed weaker synergistic effects. Thus, further mechanistic studies are warranted to confirm whether CBD piperazinyl derivatives can serve as adjuvant agents (which refers to treatments that are used to enhance the efficacy of existing chemotherapy, e.g., cisplatin, rather than serving as a primary treatment) of chemotherapeutics in cancer treatment. This observed synergy is critical given cisplatin’s known limitations, including dose-dependent toxicity and the development of resistance in many patients. Synergistic interactions can reduce the required dose of cisplatin, potentially alleviating toxic side effects while maintaining or even enhancing therapeutic efficacy. Nevertheless, this is the first study showing that the induction of piperazine to CBD can augment its antiproliferative effects and exert synergistic effects in Caov-3 cells. By combining cannabinoids with standard chemotherapy, there is potential to improve treatment outcomes, overcome resistance mechanisms, and reduce adverse effects, ultimately offering a more comprehensive approach to ovarian cancer treatment. Further studies are warranted to optimize dosing regimens and explore these synergistic interactions *in vivo* to better understand their clinical applicability.

While our study provided evidence for the cytotoxic and synergistic effects of CBD and its derivatives in ovarian cancer cells, there are several limitations that warrant further investigation. First, all experiments were conducted *in vitro* using Caov-3 ovarian cancer cells. Although *in vitro* studies offer valuable mechanistic insights, it is necessary to confirm the efficacy, bioavailability, and safety of these compounds in a physiological context using *in vivo* models. Future studies should focus on animal models to assess the pharmacokinetics of CBD derivatives and determine optimal dosing regimens. Additionally, while our findings suggest that the piperazinyl modifications to CBD enhance cytotoxicity, the specific molecular interactions and downstream effects of these modifications remain to be fully elucidated. Furthermore, future work should extend to include cisplatin-resistant ovarian cancer cell lines to evaluate whether CBD derivatives can overcome chemotherapy resistance, which is a major hurdle in ovarian cancer treatment. Future research should make efforts in these areas to support the translational potential of cannabinoid derivatives as adjuvants to conventional chemotherapy.

## 4 Conclusion

This study reports findings from the evaluation of the cytotoxic and synergistic effects of cannabinoids, particularly CBD and its synthetic derivatives, against human ovarian cancer cells (Caov-3). Among the tested natural cannabinoids, CBD exhibited the strongest antiproliferative activity and served as a promising lead compound for further investigation. The synthesis and evaluation of CBD derivatives, especially compounds **19** and **21** with a piperazinyl moiety, showed enhanced antiproliferative effects through the mediation of programmed cell death. Furthermore, compounds **19** and **21** demonstrated significant synergistic effects when combined with cisplatin, suggesting their potential to enhance the efficacy of standard chemotherapeutics. This combination strategy holds potential promise for reducing cisplatin dosage and associated toxicity, which provides insights into cannabinoids’ role as adjuvant agents in cancer treatment. However, the distinct mechanistic pathways of apoptosis and ferroptosis elicited by CBD and its derivatives underline the complexity of their action, warranting further investigation to elucidate the precise mechanisms of action. In summary, this work supports the therapeutic potential of cannabinoids, both as standalone agents and in combination with conventional chemotherapy, for the management of ovarian cancer. Future studies are warranted to explore the detailed molecular mechanisms of these compounds and assess their efficacy *in vivo* for the development of potential cannabinoid-based therapeutics in oncology.

## Data Availability

The raw data supporting the conclusions of this article will be made available by the authors, without undue reservation.

## Abbreviations

CBC, cannabichromene; CBD, cannabidiol; CBDA, cannabidiolic acid; CBDV, cannabidivarin; CBG, cannabigerol; CBGA, cannabigerolic acid; CBN, cannabinol; CBT, cannabicitran; DCF-DA, 2′,7′-dichlorofluorescin diacetate fluorescent; DMEM, Dulbecco’s modified Eagle’s medium; DMSO, dimethyl sulfoxide; MTT, 3-(4,5-dimethylthiazol-2-yl)-2,5-diphenyltetrazolium bromide; PBS, Phosphate-buffered saline; *Δ*8-THC, delta-8-tetrahydrocannabinol; ROS, Reactive oxygen species.

## References

[B1] Alsalamat H. A. Abuarab S. F. Salamah H. M. Ishqair A. H. Dwikat M. F. Nourelden A. Z. (2024). Cannabis and cancer: unveiling the potential of a green ally in breast, colorectal, and prostate cancer. J. Cannabis Res. 6, 24. 10.1186/s42238-024-00233-z 38755733 PMC11097556

[B2] Barrie A. M. Gushue A. C. Eskander R. N. (2019). Dramatic response to Laetrile and cannabidiol (CBD) oil in a patient with metastatic low grade serous ovarian carcinoma. Gynecol. Oncol. Rep. 29, 10–12. 10.1016/j.gore.2019.05.004 31193514 PMC6535622

[B3] Bijnsdorp I. V. Giovannetti E. Peters G. J. (2011). “Analysis of drug interactions,” in Cancer cell culture: methods and protocols. Editor Cree I. A. (Totowa, NJ: Humana Press), 421–434. 10.1007/978-1-61779-080-5_34 21516426

[B4] Cortez A. J. Tudrej P. Kujawa K. A. Lisowska K. M. (2018). Advances in ovarian cancer therapy. Cancer Chemother. Pharmacol. 81, 17–38. 10.1007/s00280-017-3501-8 29249039 PMC5754410

[B5] Dasari S. Njiki S. Mbemi A. Yedjou C. G. Tchounwou P. B. (2022). Pharmacological effects of cisplatin combination with natural products in cancer chemotherapy. Int. J. Mol. Sci. 23, 1532. 10.3390/ijms23031532 35163459 PMC8835907

[B6] El-Talatini M. R. Taylor A. H. Elson J. C. Brown L. Davidson A. C. Konje J. C. (2009). Localisation and function of the endocannabinoid system in the human ovary. PLOS ONE 4, e4579. 10.1371/journal.pone.0004579 19238202 PMC2640464

[B7] Frye W. J. E. Huff L. M. Dalmasy J. M. G. Salazar P. Carter R. M. Gensler R. T. (2023). The multidrug resistance transporter P-glycoprotein confers resistance to ferroptosis inducers. cdr 6, 468–480. 10.20517/cdr.2023.29 PMC10571053 37840856

[B8] Go Y. Y. Kim S. R. Kim D. Y. Chae S.-W. Song J.-J. (2020). Cannabidiol enhances cytotoxicity of anti-cancer drugs in human head and neck squamous cell carcinoma. Sci. Rep. 10, 20622. 10.1038/s41598-020-77674-y 33244087 PMC7692486

[B9] Heider C. G. Itenberg S. A. Rao J. Ma H. Wu X. (2022). Mechanisms of cannabidiol (CBD) in cancer treatment: a review. Biology 11, 817. 10.3390/biology11060817 35741337 PMC9220307

[B10] Huang M. Lu J.-J. Ding J. (2021). Natural products in cancer therapy: past, present and future. Nat. Prod. Bioprospect. 11, 5–13. 10.1007/s13659-020-00293-7 33389713 PMC7933288

[B11] Jastrząb A. Jarocka-Karpowicz I. Skrzydlewska E. (2022). The origin and biomedical relevance of cannabigerol. Int. J. Mol. Sci. 23, 7929. 10.3390/ijms23147929 35887277 PMC9322760

[B12] Jayson G. C. Kohn E. C. Kitchener H. C. Ledermann J. A. (2014). Ovarian cancer. Lancet 384, 1376–1388. 10.1016/S0140-6736(13)62146-7 24767708

[B13] Khan M. I. Sobocińska A. A. Czarnecka A. M. Król M. Botta B. Szczylik C. (2016). The therapeutic aspects of the endocannabinoid system (ECS) for cancer and their development: from nature to laboratory. Curr. Pharm. Des. 22, 1756–1766. 10.2174/1381612822666151211094901 26654588 PMC5412000

[B14] Ledvina K. R. Suelzer E. El-Alfy A. T. (2023). Delta-8-tetrahydrocannabinol: a phytocannabinoid on the rise. RPS Pharm. Pharmacol. Rep. 2, rqad031. 10.1093/rpsppr/rqad031

[B15] Li H. He H. Liu C. Akanji T. Gutkowski J. Li R. (2022). Dietary polyphenol oleuropein and its metabolite hydroxytyrosol are moderate skin permeable elastase and collagenase inhibitors with synergistic cellular antioxidant effects in human skin fibroblasts. Int. J. Food Sci. Nutr. 73, 460–470. 10.1080/09637486.2021.1996542 34719319

[B16] Li H. Puopolo T. Seeram N. P. Liu C. Ma H. (2024). Anti-ferroptotic effect of cannabidiol in human skin keratinocytes characterized by data-independent acquisition-based proteomics. J. Nat. Prod. 87, 1493–1499. 10.1021/acs.jnatprod.3c00759 38373879

[B17] Liu C. Li H. Xu F. Jiang X. Ma H. Seeram N. P. (2021). Cannabidiol protects human skin keratinocytes from hydrogen-peroxide-induced oxidative stress via modulation of the caspase-1–IL-1β Axis. J. Nat. Prod. 84, 1563–1572. 10.1021/acs.jnatprod.1c00083 33955754

[B18] Liu C. Ma H. Slitt A. L. Seeram N. P. (2020). Inhibitory effect of cannabidiol on the activation of NLRP3 inflammasome is associated with its modulation of the P2X7 receptor in human monocytes. J. Nat. Prod. 83, 2025–2029. 10.1021/acs.jnatprod.0c00138 32374168

[B19] Long H. Hu X. Wang B. Wang Q. Wang R. Liu S. (2021). Discovery of novel apigenin–piperazine hybrids as potent and selective poly (ADP-Ribose) polymerase-1 (PARP-1) inhibitors for the treatment of cancer. J. Med. Chem. 64, 12089–12108. 10.1021/acs.jmedchem.1c00735 34404206

[B20] Lyu P. Li H. Wan J. Chen Y. Zhang Z. Wu P. (2024). Bipiperidinyl derivatives of cannabidiol enhance its antiproliferative effects in melanoma cells. Antioxidants 13, 478. 10.3390/antiox13040478 38671925 PMC11047683

[B21] Ma H. Xu F. Liu C. Seeram N. P. (2021). A network pharmacology approach to identify potential molecular targets for cannabidiol’s anti-inflammatory activity. Cannabis Cannabinoid Res. 6, 288–299. 10.1089/can.2020.0025 33998855 PMC8380804

[B22] Ma L. Zhang H. Liu C. Liu M. Shangguan F. Liu Y. (2023). A novel mechanism of cannabidiol in suppressing ovarian cancer through LAIR-1 mediated mitochondrial dysfunction and apoptosis. Environ. Toxicol. 38, 1118–1132. 10.1002/tox.23752 36810933

[B23] Mangal N. Erridge S. Habib N. Sadanandam A. Reebye V. Sodergren M. H. (2021). Cannabinoids in the landscape of cancer. J. Cancer Res. Clin. Oncol. 147, 2507–2534. 10.1007/s00432-021-03710-7 34259916 PMC8310855

[B24] Misri S. Kaul K. Mishra S. Charan M. Verma A. K. Barr M. P. (2022). Cannabidiol inhibits tumorigenesis in cisplatin-resistant non-small cell lung cancer via TRPV2. Cancers 14, 1181. 10.3390/cancers14051181 35267489 PMC8909073

[B25] Montopoli M. Ragazzi E. Froldi G. Caparrotta L. (2009). Cell-cycle inhibition and apoptosis induced by curcumin and cisplatin or oxaliplatin in human ovarian carcinoma cells. Cell Prolif. 42, 195–206. 10.1111/j.1365-2184.2009.00585.x 19236381 PMC6495462

[B26] Pistollato F. Calderón Iglesias R. Ruiz R. Aparicio S. Crespo J. Dzul Lopez L. (2017). The use of natural compounds for the targeting and chemoprevention of ovarian cancer. Cancer Lett. 411, 191–200. 10.1016/j.canlet.2017.09.050 29017913

[B27] Puopolo T. Cai A. Liu C. Ma H. Seeram N. P. (2023). Investigating cannabinoids as P2X purinoreceptor 4 ligands by using surface plasmon resonance and computational docking. Heliyon 9, e21265. 10.1016/j.heliyon.2023.e21265 37920520 PMC10618793

[B28] Rathi A. K. Syed R. Shin H.-S. Patel R. V. (2016). Piperazine derivatives for therapeutic use: a patent review (2010-present). Expert Opin. Ther. Pat. 26, 777–797. 10.1080/13543776.2016.1189902 27177234

[B29] Schuel H. Burkman L. J. Lippes J. Crickard K. Forester E. Piomelli D. (2002). *N*-Acylethanolamines in human reproductive fluids. Chem. Phys. Lipids 121, 211–227. 10.1016/S0009-3084(02)00158-5 12505702

[B30] Sooda K. Allison S. J. Javid F. A. (2023). Investigation of the cytotoxicity induced by cannabinoids on human ovarian carcinoma cells. Pharmacol. Res. and Perspect. 11, e01152. 10.1002/prp2.1152 38100640 PMC10723784

[B31] Wang L. Chen X. Yan C. (2022). Ferroptosis: an emerging therapeutic opportunity for cancer. Genes and Dis. 9, 334–346. 10.1016/j.gendis.2020.09.005 PMC8843872 35224150

[B32] Whynot E. G. Tomko A. M. Dupré D. J. (2023). Anticancer properties of cannabidiol and Δ9-tetrahydrocannabinol and synergistic effects with gemcitabine and cisplatin in bladder cancer cell lines. J. Cannabis Res. 5, 7. 10.1186/s42238-023-00174-z 36870996 PMC9985258

[B33] Zhang Z. Luo Z. Sun Y. Deng D. Su K. Li J. (2023). Discovery of novel cannabidiol derivatives with augmented antibacterial agents against methicillin-resistant *Staphylococcus aureus* . Bioorg. Chem. 141, 106911. 10.1016/j.bioorg.2023.106911 37832223

[B34] Zoń A. Bednarek I. (2023). Cisplatin in ovarian cancer treatment—known limitations in therapy force new solutions. Int. J. Mol. Sci. 24, 7585. 10.3390/ijms24087585 37108749 PMC10146189

